# Butterfly Transforms for Efficient Representation of Spatially Variant Point Spread Functions in Bayesian Imaging

**DOI:** 10.3390/e25040652

**Published:** 2023-04-13

**Authors:** Vincent Eberle, Philipp Frank, Julia Stadler, Silvan Streit, Torsten Enßlin

**Affiliations:** 1 Max Planck Institute for Astrophysics, Karl-Schwarzschild-Straße 1, 85748 Garching, Germany; 2 Faculty of Physics, Ludwig-Maximilians-Universität München (LMU), Geschwister-Scholl-Platz 1, 80539 München, Germany; 3 Fraunhofer Institute for Applied and Integrated Security AISEC, Lichtenbergstraße 11, 85748 Garching, Germany

**Keywords:** response functions, spatially variant point spread functions, convolution, Bayesian imaging, butterfly matrices, Toeplitz matrices, sparse representations, neural networks

## Abstract

Bayesian imaging algorithms are becoming increasingly important in, e.g., astronomy, medicine and biology. Given that many of these algorithms compute iterative solutions to high-dimensional inverse problems, the efficiency and accuracy of the instrument response representation are of high importance for the imaging process. For efficiency reasons, point spread functions, which make up a large fraction of the response functions of telescopes and microscopes, are usually assumed to be spatially invariant in a given field of view and can thus be represented by a convolution. For many instruments, this assumption does not hold and degrades the accuracy of the instrument representation. Here, we discuss the application of butterfly transforms, which are linear neural network structures whose sizes scale sub-quadratically with the number of data points. Butterfly transforms are efficient by design, since they are inspired by the structure of the Cooley–Tukey fast Fourier transform. In this work, we combine them in several ways into butterfly networks, compare the different architectures with respect to their performance and identify a representation that is suitable for the efficient representation of a synthetic spatially variant point spread function up to a 1% error. Furthermore, we show its application in a short synthetic example.

## 1. Introduction

Images of astronomical objects are the result of measurements by physical instruments and intricate post-processing. In this procedure, instrument responses play an important role as they build the connection between the signal, i.e., the quantity of interest, and the observables.

Unfortunately, instrument responses are often non-trivial and hard to model in a simple and numerically efficient form. Examples for such instruments are the X-ray observatories eROSITA (extended ROentgen Survey with an Imaging Telescope Array) [1] and Chandra [2]. Both are challenging to compute due to their inhomogeneous behaviour in terms of space and energy. In order to efficiently perform statistical field inference, for example, by using NIFTy (Numerical Information Field Theory) [3,4,5], a Python software package for the numerical application of information field theory [6,7,8,9], these responses must be represented numerically in a way that is fast and differentiable. One promising candidate for the efficient representation of instrument responses are butterfly transforms, a linear neural network structure inspired by the structure of the fast Fourier transform (FFT) algorithm, whose size scales with O(NlogN), where N is the number of pixels.

In many cases, the measurement equation for some data *d*, taken with an instrument response *R* of the signal *s* assuming additive noise *n*, can be formulated as d=R(s)+n. Regarding photographic instruments this response *R* is a linear map that can be separated into two operations, *D* and *O*. Here, *D* describes the measurement process of the detector, while *O* represents the optical properties of the instrument. The latter is also referred to as point spread function (PSF). Since computers are used for the analysis of the experiments performed, the continuous signal space is approximated by a discrete pixelation and thus all operators can be represented as matrices.

If *O* can be approximated by a circulant matrix, a matrix consisting of cyclic permutations of the same row vector *a*, its matrix multiplication with any vector simplifies to a discrete convolution with *a*, meaning that it is spatially invariant and homogeneous, respectively. In many physically relevant cases, this homogeneity can approximately be assumed for a given observed area of the instrument. Additionally, the convolution theorem states that a convolution corresponds to a point-wise multiplication in Fourier space. Consequently, convolutional responses can be represented in an efficient way, due to the fact that one only has to store one N-entry vector instead of a $N^{2}$ matrix, as well as due to the efficiency one gains by replacing a discrete Fourier transformation by the fast Fourier transformation (FFT). Often the homogeneity assumption only holds up to a certain degree and in a limited field of view. For spatially variant PSFs, and thus non-circulant responses, efficient representations are urgently needed.

In this paper, we propose using butterfly transforms to represent spatially variant PSFs in order to build likelihoods for instruments such as eROSITA, Chandra, and many more. In particular, we present a way to parameterize butterfly transforms, combine them into networks, and compare different network architectures in terms of their efficiency and accuracy.

Section 2 summarizes the implementation and application of butterfly factorizations and transforms in previous works. Section 3 describes how butterfly transforms are parameterized in this work and how they are inspired by the structure of the Cooley–Tukey–FFT algorithm. Section 4 gives a short introduction to information field theory and Section 5 describes different designs of likelihoods. In Section 6, we define a metric in order to compare different butterfly network architectures with respect to their capability to represent the synthetic response defined in Section 7. The results, which consider a comparison of different architectures, a comparison of execution times, and a mock application of a butterfly network, can be found in Section 8.

## 2. Related Work

Butterfly factorizations and transformations are becoming increasingly popular in the machine learning community for a variety of applications. Polcari describes in [10] how the generalization of the butterfly structure known from the FFT algorithm can be used for multi-layer decomposition of unitary matrices. In [11], Dao et al. proposed a way to learn fast linear transformation algorithms using butterfly factorizations. They were able to learn several fast linear transformations, e.g., FFT, discrete sine transform, etc., and showed that their approach can be used as an efficient replacement for generic matrices in machine learning pipelines. Alizadeh et al. [12] proposed butterfly transformations as a replacement for pointwise convolutions in depth-wise separable convolutions in convolutional neural networks. This is particularly important for architectures such as MobileNets [13,14,15] that are designed to run on mobile devices. Singhal et al. [16] used complex-valued butterfly transforms for hyperspectral image processing. The combination of complex-valued multi-scale feature representation with data-driven feature learning results in lighter, yet accurate classification models. Lin et al. [17] also proposed a new form of butterfly transform, called deformable butterflies, as a replacement for convolutional or fully connected layers in neural networks. Song et al. [18] instead used the butterfly algorithm to encrypt optical images.

Our contribution to the field demonstrates the application of butterfly transforms to efficiently represent spatially varying point spread functions, necessary for accurate Bayesian imaging. A proceeding paper presented an early stage of this work [19]. In this article, however, we go more into the details of the method, theoretically address the scaling of the networks, and introduce error maps as a new visualisation method. We also perform a comparison of the execution time between a butterfly network Python implementation and a full matrix-vector multiplication. In addition, we show the behaviour of the response approximation at a higher resolution and use butterfly networks as a response representation for a synthetic Bayesian imaging task.

## 3. Methods

### 3.1. Fast Fourier Transformation

Due to the convolution theorem, Fourier transformation is one of the key elements of convolutional processes and thus the algorithm of FFT is highly relevant for the representation of instrument responses on regular grids. The main idea of the FFT is to split the sum in the discrete Fourier transform (DFT),

(1) $${\hat{f}}_{k}=\frac{1}{\sqrt{N}}\sum_{x=0}^{N-1}f_{x}\;\cdot \;e^{-2\pi i\;\cdot \;\frac{kx}{N}}\;,\;$$

into two sums, over even and odd indices [20]. By using the mathematical properties of the N-th primitive root $\omega_{N}=e^{\frac{-2\pi i}{N}}$, it can be shown that

(2) $$\begin{array}{cc} {\hat{f}}_{k} & =\frac{1}{\sqrt{N}}\sum_{\xi =0}^{N/2-1}\omega_{N}^{k(2\xi )}f_{2\xi }+\frac{1}{\sqrt{N}}\sum_{\xi =0}^{N/2-1}\omega_{N}^{k(2\xi +1)}f_{2\xi +1}\\ \; & =\frac{1}{\sqrt{2}}\left[\frac{1}{\sqrt{\frac{N}{2}}}\sum_{\xi =0}^{N/2-1}\omega_{\frac{N}{2}}^{k\xi }f_{2\xi }\right]+\frac{1}{\sqrt{2}}\omega_{N}^{k}\left[\frac{1}{\sqrt{\frac{N}{2}}}\sum_{\xi =0}^{N/2-1}\omega_{\frac{N}{2}}^{k\xi }f_{2\xi +1}\right]\\ \; & =\frac{1}{\sqrt{2}}{\hat{f}}_{k}^{\;\mathrm{even}}+\frac{1}{\sqrt{2}}\omega_{N}^{k}{\hat{f}}_{k}^{\;\mathrm{odd}}\;. \end{array}$$

Taking a closer look at the Fourier component ${\hat{f}}_{k+\frac{N}{2}}$ shows that one can reuse the same two components, ${\hat{f}}_{k}^{\;\mathrm{even}}$ and ${\hat{f}}_{k}^{\;\mathrm{odd}}$, which were already calculated:

(3) $$\begin{array}{cc} {\hat{f}}_{k+\frac{N}{2}} & =\frac{1}{\sqrt{N}}\sum_{\xi =0}^{N/2-1}\omega_{N}^{\left(k+\frac{N}{2}\right)\left(2\xi \right)}f_{2\xi }+\frac{1}{\sqrt{N}}\sum_{\xi =0}^{N/2-1}\omega_{N}^{\left(k+\frac{N}{2}\right)\left(2\xi +1\right)}f_{2\xi +1}\\ \; & =\frac{1}{\sqrt{2}}\left[\frac{1}{\sqrt{\frac{N}{2}}}\sum_{\xi =0}^{N/2-1}\omega_{N}^{N\xi }\omega_{\frac{N}{2}}^{k\xi }f_{2\xi }\right]+\frac{1}{\sqrt{2}}\omega_{N}^{k+\frac{N}{2}}\left[\frac{1}{\sqrt{\frac{N}{2}}}\sum_{\xi =0}^{N/2-1}\omega_{N}^{N\xi }\omega_{\frac{N}{2}}^{k\xi }f_{2\xi +1}\right]\\ \; & =\frac{1}{\sqrt{2}}{\hat{f}}_{k}^{\;\mathrm{even}}+\frac{1}{\sqrt{2}}\omega_{N}^{k+\frac{N}{2}}{\hat{f}}_{k}^{\;\mathrm{odd}}\\ \; & =\frac{1}{\sqrt{2}}{\hat{f}}_{k}^{\;\mathrm{even}}+\frac{1}{\sqrt{2}}\omega_{N}^{\frac{N}{2}}\omega_{N}^{k}{\hat{f}}_{k}^{\;\mathrm{odd}}\\ \; & =\frac{1}{\sqrt{2}}{\hat{f}}_{k}^{\;\mathrm{even}}-\frac{1}{\sqrt{2}}\omega_{N}^{k}{\hat{f}}_{k}^{\;\mathrm{odd}}\;. \end{array}$$

This means that an N-sized Fourier transform can be separated into two N/2-sized Fourier transforms along the even and odd indices, also called the Danielson–Lanczos Lemma found in 1942 [21]. The components ${\hat{f}}_{k}^{\;\mathrm{even}}$ and ${\hat{f}}_{k}^{\;\mathrm{odd}}$ can then be used to calculate $f_{k}$ and $f_{k+\frac{N}{2}}$. Putting together the relations in Equations (2) and (3) yields

(4) $$\left(\begin{array}{c} {\hat{f}}_{k}\\ {\hat{f}}_{k+N/2} \end{array}\right)=\frac{1}{\sqrt{2}}\left(\begin{array}{cc} 1 & 1\\ 1 & -1 \end{array}\right)\left(\begin{array}{c} {\hat{f}}_{k}^{\;\mathrm{even}}\\ \omega_{N}^{k}{\hat{f}}_{k}^{\;\mathrm{odd}} \end{array}\right)\;.\;$$

The two smaller Fourier transforms can be separated in the same way, resulting in a divide and conquer algorithm. Assuming that the initial value of N is a power of 2, this splitting can be applied $\log_{2}\left(N\right)$ times. Inspired by machine learning language, these iterations are called layers in the following. With N additions in each of these layers, the total computational complexity is about $O(N\log_{2}N)$. Comparing this to a regular DFT with its computational complexity of $O(N^{2})$ (N components with N summands) the amount of saved time in the FFT algorithm is significant.

Due to the layer-wise splitting into even and odd indices one has to spend some additional effort on renumbering and book-keeping in order to combine the correct indices. In 1965, Cooley and Tukey discovered that this iterative splitting into even and odd indices can be represented as a bit reversal of the indices (reading backwards in binary representation) [21]. Therefore, one does not have to take additional care of the right input ordering. Besides the “decimation in time algorithm” by Cooley and Tukey, there are other FFT algorithms that are not further considered in this work.

### 3.2. Butterfly Transform and Convolution

**Figure 1:**
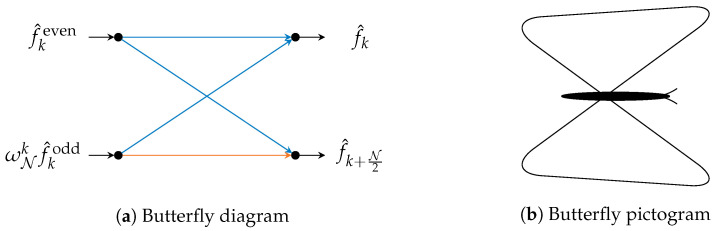
Comparison of: (**a**) a butterfly diagram—blue lines indicate an addition, the orange line indicates subtraction; (**b**) pictogram of a butterfly with similar appearance.

The data-flow diagram illustrating the algorithm of Equation (4) is often called a butterfly diagram because of its resemblance to a butterfly (see Figure 1). Here, the direction and color of the arrows pointing to a node describe the mathematical operations being performed. Since the abstraction of the FFT algorithm results in a similar data flow diagram, it will be called the butterfly transform in the following. As the butterfly diagrams always connect to two components, most of the descriptions used in the following, concerning their parameterization, are two-dimensional to keep the notation simple.

In order to generalize the FFT while preserving its efficient structure, we decompose the operations in Equation (4) into a diagonal operator Φ and a mixing operator Θ, as given in the following.

(5) $$\Phi =\left(\begin{array}{cc} 1 & 0\\ 0 & \omega_{N} \end{array}\right),\;\Theta =\frac{1}{\sqrt{2}}\left(\begin{array}{cc} 1 & 1\\ 1 & -1 \end{array}\right),$$

and thus

(6) $$\left(\begin{array}{c} {\hat{f}}_{k}\\ {\hat{f}}_{k+N/2} \end{array}\right)=\Theta \;\Phi \left(\begin{array}{c} {\hat{f}}_{k}^{\;\mathrm{even}}\\ {\hat{f}}_{k}^{\;\mathrm{odd}} \end{array}\right)\;.\;$$

For each component, we introduce free parameters that control how the operation deviates from an ordinary FFT. A general representation of Θ is obtained by parameterizing it by the sine and cosine of an angle θ,

(7) $$\Theta_{\theta }=\left(\begin{array}{cc} \cos\theta & \sin\theta \\ \sin\theta & -\cos\theta \end{array}\right)\;.\;$$

To preserve the generality of the transformation within one layer, the θs for different connected pairs, denoted by the index *k* in Equation (6), are independent. This means that for an N-size transformation there are N/2θs, in each layer, regulating the interaction between two connected data points. Considering this parameterization, we obtain the Θ from Equation (5), i.e., the one for an FFT, by inserting $\theta =\frac{\pi }{4}$. The operator Φ is parameterized as  

(8) $$\Phi_{\phi }=\left(\begin{array}{cc} e^{i\phi_{1}} & 0\\ 0 & e^{i\phi_{2}} \end{array}\right)\;,\;\phi_{j}\in R\;.$$

This parameterization makes it possible to recover the correct phases for an FFT, but also to change them in an arbitrary way. The combination of Θ and Φ is sufficient to represent an entire FFT transform. To obtain an even more general transformation, the diagonal operator Γ,

(9) $$\Gamma_{\gamma }=\left(\begin{array}{cc} e^{\gamma_{1}} & 0\\ 0 & e^{\gamma_{2}} \end{array}\right)\;,\;\gamma_{j}\in R\;,$$

is introduced, which accounts for the real-valued amplitudes. This leads to a loss of unitarity for $\gamma_{1},\;\gamma_{2}\neq 0$ in the combined transformation of Γ, Φ and Θ.

Now, we can build a generic butterfly-structured transformation *B*, using the layered structure of an FFT as a guiding example. The subscript of the operators refers to the layer in the FFT algorithm and thus implies that the correct components are connected.

(10) $$B=\Gamma_{0}\Phi_{0}\Theta_{0}\ldots \Gamma_{j}\Phi_{j}\Theta_{j}\ldots \Gamma_{n-1}\Phi_{n-1}\Theta_{n-1}\;.$$

Given this butterfly transformation and the structure of a convolution operation, based on the convolution theorem, a butterfly convolution-like operator *O* can be formulated as

(11) $$O=B^{†}\Lambda B\;.$$

In this equation the Λ operator corresponds to the Fourier transformed PSF. Usually, physically reasonable PSFs are real-valued in position space and thus complex-valued in harmonic space. Therefore, the Λ operator is defined as a diagonal operator, with complex values,

(12) $$\Lambda_{\lambda }=\left(\begin{array}{cc} e^{\lambda_{1}} & 0\\ 0 & e^{\lambda_{2}} \end{array}\right)\;,\;\lambda_{j}\in C\;.$$

$B^{†}$, in Equation (11), denotes the adjoint of *B*. For some experiments, the parameters of *B* and $B^{†}$ were strictly coupled, called mirrored architecture in the following. For others, the parameters were independent, denoted by different indices, resulting in a non-mirrored architecture:

(13) $$O=B_{1}^{†}\Lambda B_{2}\;.$$

### 3.3. Multidimensional Butterfly Transformation

Since the butterfly structure is strongly related to the FFT, it would make sense to treat multidimensional butterfly transformations in the same way as multidimensional Fourier transformations. Therefore, butterfly transformations can be applied to each dimension separately. However, in this work the 2D application is slightly modified, in a way that the mixing operator Θ is applied to each axis separately (for the first axis all columns are transformed with the same θs, whereas for the second axis all row transformations share the same θs), but after this axis-wise Θ-transformation the operators Φ and Γ are applied as diagonal operators. Applying a 2D butterfly transform on a m×l 2D grid with m>l results in $\log_{2}m$ layers for the first axis transformation and $\log_{2}l$ layers for the second axis. The number of θs in one $\Theta_{i}$ operator is then $\frac{m}{2}$ and $\frac{l}{2}$, respectively. The operators Φ and Γ are then applied in $\log_{2}m$ layers. Here, the number of ϕs and γs in one $\Phi_{i}$ or $\Gamma_{i}$ operator is ml.

Another approach, next to the 2D application, is to reduce the number of dimensions to one (in this case, as we are dealing with images, from 2D to 1D) and just perform one butterfly transform to this one dimension. For the case of two-dimensional inputs the dimensionality reduction can be easily performed by concatenating all the column vectors to one long vector, which will be called flattening from now on. These two different approaches differ in the number of layers needed by the butterfly algorithm as well as in the number of parameters per layer. For a one-dimensional transform of a ml 1D grid, one obtains $\log_{2}ml$ layers. Here, the number of parameters behaves differently as there are $\frac{ml}{2}$ in each $\Theta_{i}$. The number of parameters in one $\Phi_{i}$ or $\Gamma_{i}$ is again ml.

Since in the latter case this results in a larger number of parameters, the 1D flattened transform is expected to be more flexible than the 2D transform. A more detailed listing of the number of parameters for each approach is shown in Table 1.

**Figure 2:**
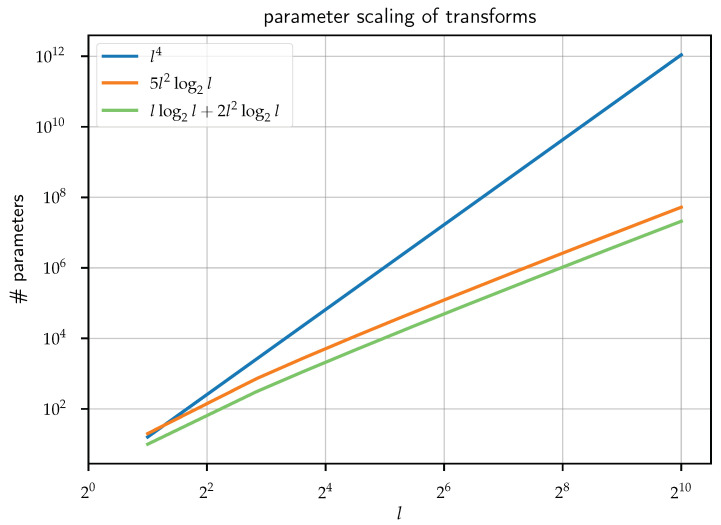
Scaling behaviour of a 2D transform represented by a full matrix representation (blue line), a 2D butterfly transform (orange line), and a 1D butterfly transform with flattened input (green line).

Their different scaling behaviour can be easily compared by setting m=l. Then the total number of parameters is $\left[l\log_{2}l+2l^{2}\log_{2}l\right]$ for the 2D and $\left[5l^{2}\log_{2}l\right]$ for the 1D flattened case. Figure 2 shows the different scaling behaviours of these two butterfly applications and of a full matrix representation as a function of the axis length *l*.

## 4. Information Field Theory

To reach a better understanding for the area of use for the efficient responses, a brief introduction to information field theory (IFT) [9] will be given. Information field theory is the application of information theory to physical fields. Probably the most important relation within information theory is Bayes’ theorem,

(14) $$P\left(s|d\right)=\frac{P(d,s)}{P(d)}=\frac{P(d|s)P(s)}{P(d)}\;,$$

which connects a posterior with the likelihood, the prior, and the evidence. Here, the likelihood P(d|s) describes how likely it is to record specific data for a given signal, which incorporates knowledge about the instrument and the measurement process. The prior P(s) is chosen with respect to the physical knowledge one has about the observed quantity or situation. The evidence P(d)=∫dsP(d|s)P(s) is needed for the proper normalization of the posterior P(s|d). Another important quantity is the information Hamiltonian that is defined as the negative logarithm of the probability, H(d,s)=−ln[P(d,s)]. Due to the properties of the logarithm and the product rule of probabilities the information Hamiltonian, H is an additive quantity H(d,s)=H(d|s)+H(s).

The likelihood can be computed from the noise statistic P(n|s) and the measurement equation, here in the form P(d|s,n)=δ(d−R(s)−n). Thus, the likelihood is

(15) $$\begin{array}{cc} P(d|s) & =\int dn\;P(d|s,n)P(n|s)\\ \; & =\int dn\;\delta (d-R(s)-n)P(n|s)=P(n=d-R(s)|s)\;. \end{array}$$

Assuming Gaussian priors for signal, G(s,S), and noise, G(n,N), and using Equation (15) the Hamiltonians simplify to

(16) $$\begin{array}{cc} H(s) & =-\ln\left[G\left(s,S\right)\right]=-\ln\left[\frac{1}{\sqrt{2\pi S}}\exp\left(-\frac{1}{2}s^{†}S^{-1}s\right)\right]\\ \; & =\frac{1}{2}\ln\left|2\pi S\right|+\frac{1}{2}s^{†}S^{-1}s\;,\\ H(d|s) & =-\ln[G(n,N)]\\ \; & =\frac{1}{2}\ln\left|2\pi N\right|+\frac{1}{2}{\left(d-R\left(s\right)\right)}^{†}N^{-1}\left(d-R\left(s\right)\right)\;. \end{array}$$

However, if the measurement process follows Poisson statistics, which is the case for realistic photographic measurements, a Poissonian likelihood model has to be used. For this, we define the event density field *s* at a sky coordinate *x* as $s^{x}$ and the density of observed events in a detector bin *i* as $\mu^{i}$. The connection between the event density and the observed event density is described by the instrument response $R_{\;x}^{i}$ in the equation

(17) $$\mu^{i}=R_{\;x}^{i}s^{x}\;.$$

*R* thus describes the optical properties of the instrument and the detector. For the case that all measured events are independent of each other, the likelihood P(d|μ) follows a Poisson distribution

(18) $$P\left(d|\mu \right)=\prod_{i=1}^{N}\frac{{\left(\mu^{i}\right)}^{d^{i}}e^{-\mu^{i}}}{d^{i}!}$$

and thus, following the definition of the information Hamiltonian, the likelihood Hamiltonian is

(19) $$H\left(d|\mu \right)=\sum_{i=1}^{N}\left[\mu^{i}-d^{i}\ln\mu^{i}+\ln\left(d^{i}!\right)\right]\;.$$

For a Poisson distribution the density *s* has to be strictly positive. Thus, the prior P(s) can no longer be Gaussian, but must follow a strictly positive distribution, e.g., a log-normal distribution or an inverse gamma distribution. Dropping all the constant terms, symbolized by $\hat{=}$ meaning “up to constants in any here relevant quantity”, the likelihood Hamiltonian can be formulated as

(20) $$H\left(d|s\right)\hat{=}\sum_{i=1}^{N}\left[R_{\;x}^{i}s^{x}-d^{i}\ln\left(R_{\;x}^{i}s^{x}\right)\right]$$

One way to find an estimate for the signal *s* is to maximize the probability P(s|d) by minimizing the joint Hamiltonian H(d,s), with respect to the signal *s*. This is the maximum a posteriori (MAP) approximation. Other inference methods including an uncertainty quantification are metric Gaussian variational inference (MGVI) [22] or geometric variational inference (geoVI) [23]. As a minimization algorithm, Newton-CG [24] was used throughout all experiments.

## 5. Parallel and Serial Likelihoods

Models for inference processes in NIFTy are built in a forward way, as so-called generative models. This means that a model of the physical signal is created first, followed by the instrument response. Applying the IFT formalism, described in Section 4, to a generative model with a butterfly convolution operator as a response yields a likelihood with dependencies on the signal *s* and the response parameters θ, ϕ, γ, and λ.

In addition to being able to use butterfly convolution operators with mirrored, non-mirrored, flat, and 2D configurations, they can be combined into a network built in parallel or in series. In the case where *n* multiple butterfly convolution operators are connected in series, the response operator representation in Equation (16) or (20) arises from the sequential application of multiple butterfly convolution operators,

(21) $$R\left(s,\theta ,\phi ,\gamma ,\lambda \right)=O_{1}\ldots O_{n}s\;.$$

In contrast, with *n* butterfly convolution operators arranged in a parallel architecture, each butterfly convolution operator is applied to the signal, and the results are summed. The corresponding instrument response representation is then

(22) $$R\left(s,\theta ,\phi ,\gamma ,\lambda \right)=\left(O_{1}+\cdots +O_{n}\right)s\;.$$

Before using a particular butterfly network as an instrument response function in an imaging application, it is trained on signal-data pairs of the instrument. For many instruments, specific knowledge of the PSFs is available in the form of signal-data pairs, either from an expensive one-time simulation or from extrapolation of calibration measurements. Using these signal-data pairs, the joint Hamiltonian H(d,s,θ,ϕ,γ,λ) is minimized with respect to the response parameters θ, ϕ, γ, and λ, resulting in a MAP approximation of the instrument. The initial values for these parameters, $\tilde{\theta },\tilde{\phi },\tilde{\gamma }$, and $\tilde{\lambda }$, are chosen such that all $O_{i}$ correspond to a convolution with a delta peak ($\tilde{\theta }=\pi /4$, $\tilde{\gamma }=0$, $\tilde{\lambda }=1$, and $\tilde{\phi }$ according to the needed phases, see Section 3.1). The prior distribution of the parameters is assumed to be Gaussian, with means at the initial values and unit variance. The final goal of the minimization is to obtain an efficient digital twin of the real physical instrument.

Once a butterfly response is trained, it can be used for imaging with the corresponding instrument. For this, the response parameters are fixed to the inferred values $\underline{\theta },\underline{\phi },\underline{\gamma }$, and $\underline{\lambda }$, resulting in a response operator, which is linear in the signal *s*. The selection of a suitable generative model for *s* depends on the observation of interest. In order to obtain an uncertainty estimate for the physical signal *s*, the inference algorithms MGVI or geoVI can be used.

## 6. Evaluation of the Response Approximation

Before using a trained butterfly response in an inference algorithm, it must be certified that the mapping performed by the response representation is sufficiently accurate. Therefore, we compare the action caused by a signal, here a point source at position *z*, s(x)=δ(x−z), of the to-be-learned or simulated response with the butterfly response by their difference. This will be called the response approximation error

(23) $$E\left(s\right)=R_{\;\mathrm{sim}.}\left(s\right)-R_{\;\mathrm{but}.}\left(s\right)\;.$$

To keep the evaluation simple, unit brightness point sources at all signal domain locations z∈Ω are considered. In the next step, the 2-norm (${∥s∥}_{2}=\sqrt{\sum_{x\in \Omega }{\left|s_{x}\right|}^{2}}$) of E(s) is calculated for all these sources individually and normalized by the 2-norm of the corresponding true signal response:

(24) $$\hat{\epsilon_{z}}=\frac{{∥E\left[\delta \left(x-z\right)\right]∥}_{2}}{∥R_{\mathrm{sim}.}{\left[\delta \left(x-z\right)\right]∥}_{2}}=\frac{∥R_{\;\mathrm{sim}.}\left[\delta \left(x-z\right)\right]-R_{\;\mathrm{but}.}{\left[\delta \left(x-z\right)\right]∥}_{2}}{∥R_{\mathrm{sim}.}{\left[\delta \left(x-z\right)\right]∥}_{2}}\;,$$

where $\hat{\epsilon_{z}}$ defines a relative error for each pixel *z* and thus results in an image, called the error map, in the following. This error map depicts the errors introduced by the butterfly response in total and shows which areas are more reliable and in which areas the mapping deviates significantly from the approximated response.

In order to quantify the total error with respect to all mapping errors, we calculate the 2-norm of the 4D matrix $E_{z}=E\left[\delta \left(x-z\right)\right]$, containing the error images for all possible z-values and normalize it by dividing with the 2-norm of the matrix $R_{z}=R_{\mathrm{sim}.}\left[\delta \left(x-z\right)\right]$, containing all true simulated responses resulting in the the total error $\hat{\zeta }$:

(25) $$\hat{\zeta }=\frac{{∥E∥}_{2}}{∥R_{\mathrm{sim}.}{∥}_{2}}\;.$$

## 7. Synthetic Response

In order to investigate whether and to what degree butterfly networks are capable of approximating spatially variant PSFs, they were trained to approximate a synthetic response. This synthetic response can be regarded as the convolution of the signal *s*, which is a point source located at the position *z*, s(x)=δ(x−z), with a rotational symmetric PSF with a position-dependent shape,

(26) $${\left(Rs\right)}^{y}=\int_{\Omega }\mathrm{PSF}\left(y-x,x\right)s\left(x\right)\;dx\;.$$

For the PSF a zero-centred Gaussian was chosen,

(27) $$\mathrm{PSF}\left(x,z\right)=G\left(\rho ,\sigma^{2}\right)=\frac{1}{\sqrt{2\pi \sigma^{2}}}\exp\left(-\frac{\rho^{2}}{2\sigma^{2}\left(z\right)}\right)\;,$$

with $\rho ={∥x∥}_{2}$, where *x* is the coordinate vector of the image plane and ${∥x∥}_{2}=\sqrt{x_{1}^{2}+x_{2}^{2}}$ is its length. The dependence on the position *z* of the point source is encoded in the variance $\sigma^{2}\left(z\right)$ of the Gaussian. To keep this spatial dependency simple, only the distance from the centre of the image *c* to the point source *z*, $r={∥c-z∥}_{2}$, influences the shape of the PSF. As this absolute value depends on the image resolution, *r* will be normalized by the maximal distance within the image, $\hat{r}=r/r_{\max}$, to obtain a relative measure for the distance being in the interval [0,1]. As indicated, the variance $\sigma^{2}$ is a function of this relative distance $\hat{r}$ between the point source at *z* and the image centre *c*,

(28) $$\sigma^{2}\left(\hat{r}\right)=\beta \;\cdot \;{\hat{r}}^{2}+\eta \;.$$

**Figure 3:**
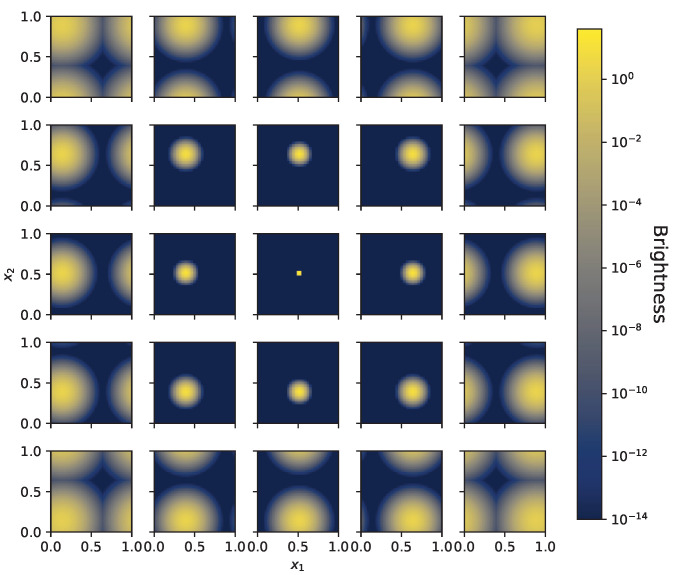
25 signal responses R(s) for point sources at different positions. For simplicity we used periodic boundaries for the kernels, which will be properly addressed in the future. The colours show the resulting brightness values.

The two parameters are set to β=0.01 and $\eta =10^{-5}$. Following Equation (28), larger distances $\hat{r}$ lead to larger values of the variance $\sigma^{2}$. This means that point sources with smaller values of $\hat{r}$ are convolved with a sharper Gaussian, while point sources at further distances from the centre are convolved with broader Gaussians (see Figure 3). This results in an spatially variant PSF, which can be used to examine the expressiveness of the butterfly architecture.

## 8. Results

### 8.1. Comparison of Architectures

In search of a butterfly network capable of representing spatially variant point spread functions, various architectures were compared, in terms of their ability to represent the synthetic response, differing in their number of butterfly convolution operators (BCOs), mirrored (mr) or non-mirrored (nmr) architecture, flat or 2D network design, and serial or parallel built likelihood (see Table 2). All of these networks were trained to approximate the synthetic response described in Section 7 by maximizing the posterior (MAP) until the optimization was sufficiently converged (300 Newton steps). As training data, a set of all possible PSFs within the given pixelation of 16×16 was used. The signals were fixed to be point sources with brightness values of 40 at the corresponding positions. A Gaussian likelihood was used and the noise covariance *N* was set to be diagonal with entries of $10^{-6}$. In order to gain a better understanding of the influence of some of these properties on the total approximation behaviour, the networks are regarded separately and with respect to their final total approximation error $\hat{\zeta }$ in Table 2.

The comparison of the $\hat{\zeta }$ value of ${\mathrm{Net}}_{1}$, ${\mathrm{Net}}_{2}$, and ${\mathrm{Net}}_{3}$ with 1, 2, and 3 BCOs, but otherwise the same properties, shows that a higher number of BCOs lowers the total error and thus increases the approximation capability. The second property of interest is the kind of architecture used, mirrored or non-mirrored. Therefore the $\hat{\zeta }$ value of ${\mathrm{Net}}_{3}$, with its mirrored architecture, is compared to the one of ${\mathrm{Net}}_{4}$, with its non-mirrored architecture, while their other properties are equivalent. This shows that the non-mirrored architecture performs better than the mirrored one. The same conclusion can be drawn by comparing $\hat{\zeta }$ of ${\mathrm{Net}}_{5}$ and ${\mathrm{Net}}_{6}$, which also only differ in their state of mirroring. In a similar way the flattened and 2D applications can be examined. Since ${\mathrm{Net}}_{3}$ and ${\mathrm{Net}}_{5}$ only differ in this property, their error values suggest that the flat application is superior to the 2D application with respect to the reconstruction capability. This is confirmed regarding the error of ${\mathrm{Net}}_{4}$ and ${\mathrm{Net}}_{6}$, which are in a similar relationship.

Since more BCOs, flattening, and a non-mirrored architecture increase the number of parameters and thus lead to more degrees of freedom, it is assumed that these architectures are more flexible and can approximate the true response in a better way.

**Figure 4:**
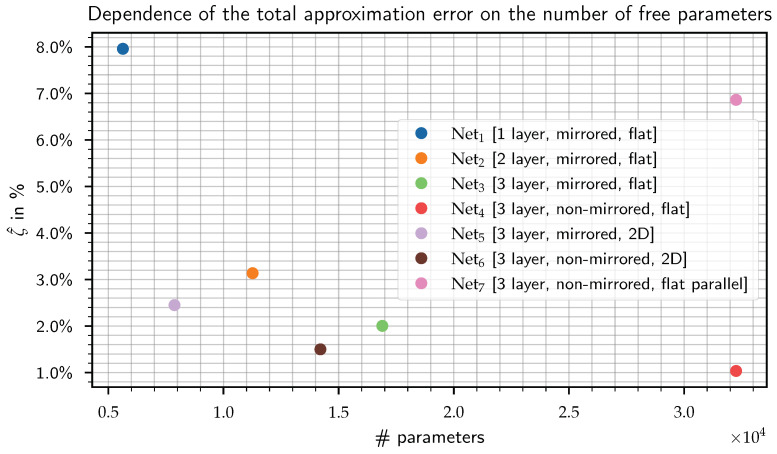
Total approximation error $\hat{\zeta }$ with respect to the number of parameters in the network. A combination of low error and a low number of parameters is important for a good efficiency of the corresponding network.

For the overall efficiency of the various networks it is not only important to approximate the synthetic response in a optimal way, but also to keep the number of parameters, and thus the network density (network density is defined here as the ratio of network parameters and number of entries in a full matrix representation), as low as possible (see Figure 4). In the examined cases, sparser architectures tend to perform worse in comparison to architectures with more parameters. Overall ${\mathrm{Net}}_{4}$ approximates the synthetic response best with an 1% error. ${\mathrm{Net}}_{6}$, however, has only 44% of the parameters of ${\mathrm{Net}}_{4}$ and is therefore less dense. This goes hand in hand with a slightly increased approximation error by an absolute value of 0.46% (see Table 2). In the end, the number of parameters of the butterfly networks still scales with O(NlogN). This means that they become less dense with increasing resolution.

### 8.2. Performance at Increased Resolution

Although the performance of the much sparser network ${\mathrm{Net}}_{6}$ is only decreased by 0.46% in absolute error, ${\mathrm{Net}}_{4}$ is still chosen as the best performing architecture for further discussion. In the following, we will investigate how the performance of the network depends on the resolution. Therefore, a ${\mathrm{Net}}_{4}$ architecture was again trained on a complete set of signal responses of all possible point source positions with an increased resolution of 32×32. The noise covariance was not changed.

The results for 16×16 and 32×32 for the otherwise same butterfly network architecture are collected in Table 3. One can see that the approximation capability does not suffer, while the density decreases with increasing resolution. The density decreases because the number of butterfly network parameters scales with about NlogN whereas a full matrix representation would scale with $N^{2}$. This is the expected behaviour described in Section 3.3.

**Figure 5:**
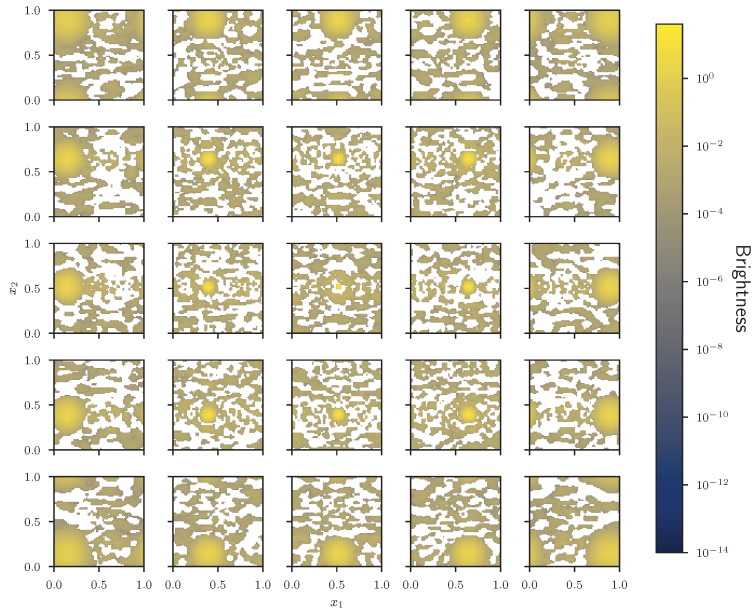
Approximated signal responses of the 25 point sources. The brightness values are shown in colour. The colourbar corresponds to the one in Figure 3.

**Figure 6:**
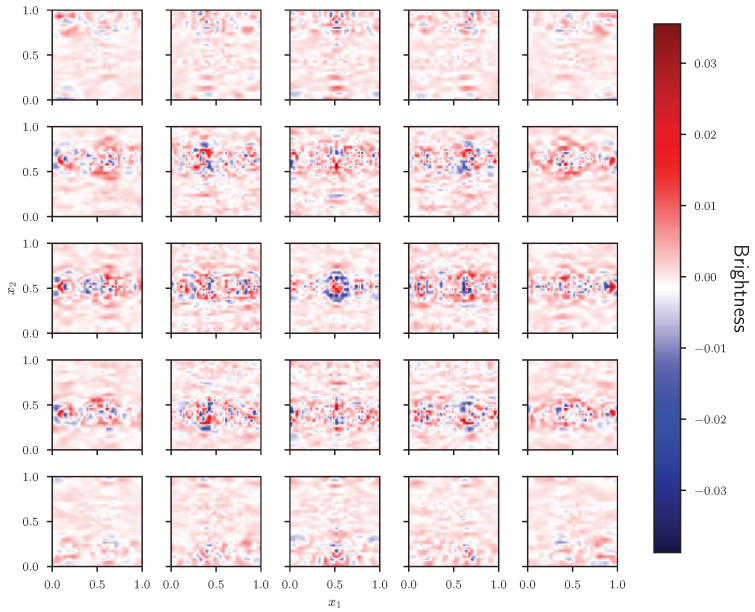
Error pictures E(s(x)=δ(x−z)) for 25 different point source positions at various positions *z*. The differences between the simulated and approximated signal responses, are shown in colour.

In order to further validate the approximation of the mapping, one can compare the synthetic signal responses (see Figure 3) and the approximated butterfly response (see Figure 5) for the same 25 different point sources. One can see that the main features, the position, the size and the magnitude of the Gaussian are picked up by the network, but also that there are not defined areas in the logarithmic plot. This is due to negative regions in the butterfly signal response caused by lack of constraints on positivity in the non-mirrored architecture. For a closer investigation we also plotted the difference between the two signal responses (see Figure 6). As presumed, the peaks of the error pictures E(s) are on the same magnitude as the total error $\hat{\zeta }$. Since these are just a few samples for the mapping, which are not representative for the overall approximation, we also consider the error maps $\hat{\epsilon }$, described in Section 6, for further investigation of the errors made by the butterfly networks.

**Figure 7:**
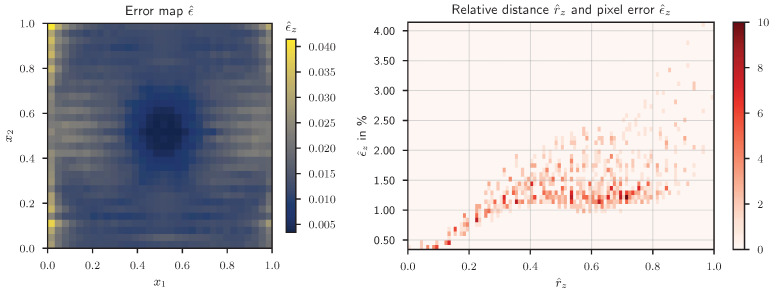
(**left**): Error map resulting from the relative error ${\hat{\epsilon }}_{z}$ for each pixel. This figure shows which areas of the butterfly response representation are more reliable than others. (**right**): 2D histogram of the relative distance ${\hat{r}}_{z}$ from the centre of the image versus the pixel error ${\hat{\epsilon }}_{z}$.

Figure 7 shows that the relative error ${\hat{\epsilon }}_{z}$ is not constant over the image domain, but smaller in the centre than at its borders. Since the only property changing with the relative distance $\hat{r}$ from the image centre is the shape and size of the synthetic response, it appears that wider PSFs are more difficult to approximate than smaller ones. This is also visualized by showing the relationship between ${\hat{r}}_{z}$ and ${\hat{\epsilon }}_{z}$ for each pixel of the error map in a 2D histogram (see right plot Figure 7).

### 8.3. Comparison of Execution Times and Memory Consumption

In addition to measuring the ability to represent the true inhomogeneous point spread function, we want to test the speed of the butterfly network implementation in an application. Therefore, we use the Python package timeit to measure the execution time of a forward pass in seconds. Here, we compare the execution time of a butterfly convolution with fixed θ,ϕ,γ, and λ against a full matrix-vector product performed by numpy.matmul. Since the numpy.matmul implementation runs on multiple cores and the butterfly network implementation is not parallelized, we force the code to run on a single core to make the numbers more comparable. [OMP_NUM_THREADS = 1, OPENBLAS_NUM_THREADS = 1, MKL_NUM_THREADS = 1]

**Figure 8:**
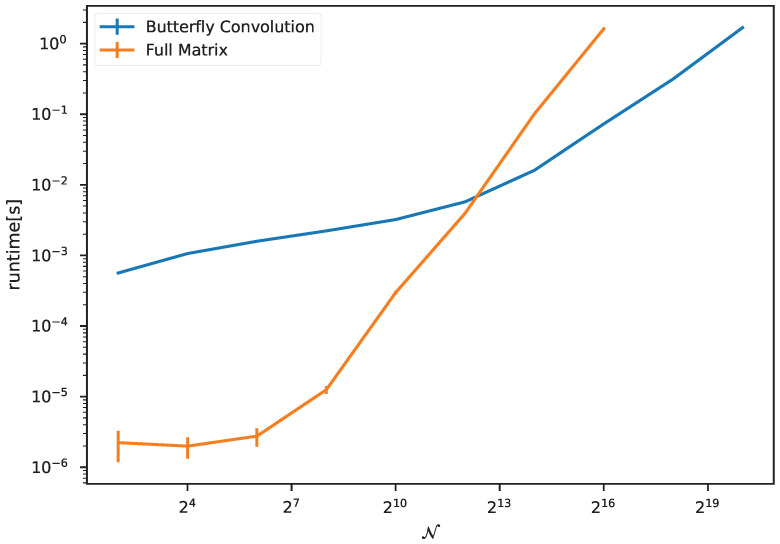
Number of pixels versus execution time in [s]. Tested on the following hardware: AMD Ryzen 7 4800H (CPU-Model), 1400 MHz (Clocking Speed), 1 Core, 64 GB (RAM).

Figure 8 and Table 4 show that for small N the butterfly convolution implementation is slower than full matrix-vector multiplication. The reason for this behaviour is that the butterfly convolution is mostly written in Python and thus has a huge overhead, whereas the matrix-vector multiplication is written in C and is highly optimised. Implementing the butterfly convolution in C should increase its speed significantly. However, this changes at higher resolution, where the butterfly convolution is considerably faster than the full matrix-vector mutliplication, despite being implemented in a slower language. This is due to the different scaling of the number of operations involved and their parameters. Since the number of parameters in a full matrix multiplication scales with $N^{2}$, it was not possible to store the matrix on a laptop for N>(256×256). This memory consumption further illustrates the importance of this method for high resolution imaging with spatially varying point spread functions.

### 8.4. Imaging with a Butterfly Response

Since the previous sections show that the efficient representation of instrument responses using butterfly networks is indeed possible up to a certain representation error, this section presents a small imaging application with a trained butterfly network and a synthetic generated data set.

**Figure 9:**
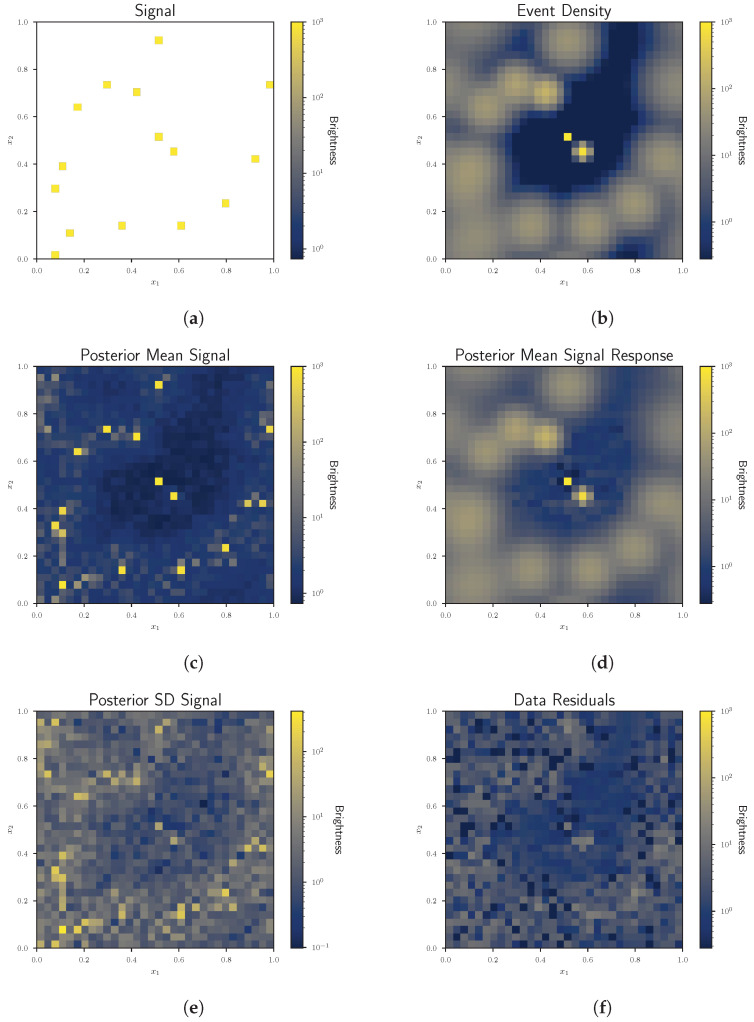
Imaging with a trained butterfly response representation: (**a**) synthetic signal, also called ground truth; (**b**) simulated observed event density; (**c**) posterior mean signal; (**d**) posterior mean signal response; (**e**) posterior standard deviation signal (**f**) the absolute difference between the data and the posterior mean signal response, called data residuals.

As already explained in Section 5, a forward model is built in order to infer a signal from collected data. To keep the validation simple, a synthetic signal consisting of 15 point sources with random positions in the signal domain and brightness values between 900 and 1000 is generated, also called ground truth (see Figure 9a) in the following. The corresponding observed event density is then calculated from this ground truth using the synthetic response from Section 7 (see Figure 9b). From this observed event density the synthetic data is generated by drawing from Poisson distributions with the respective rates.

As a model for the prior distribution P(s) of the point sources an inverse gamma distribution, $f\left(x;q,\alpha \right)=\frac{q^{\alpha }}{\Gamma (\alpha )}x^{-\alpha -1}\exp(-\frac{q}{x})$, is chosen with α=1 and q=1. For the response operator in our Poissonian likelihood we use the butterfly network ${\mathrm{Net}}_{4}\left(32x32\right)$ that was previously trained on the synthetic response, as described in Section 8.2. In order to ensure positivity in the signal response domain, we clip the signal response at a minimum of $10^{-8}$. Positivity is a necessary property here for the Poisson likelihood to be well-defined. Applying the IFT formalism, the joint Hamiltonian H(s,d) is constructed using these prior statistics and the Poissonian likelihood. For the inference we use the geoVI algorithm [23] with four mirrored samples. After inference with geoVI, we obtain a set of samples that represent the posterior distribution of the quantity of interest, in this case the signal. From these samples, we can calculate the posterior mean and standard deviation of the signal and the signal response. Comparing the posterior mean of the signal (see Figure 9c) to the ground truth (see Figure 9a), one can see that most of the signal is sufficiently reconstructed. The positions and brightness values in the centre are reconstructed very well, whereas there are some errors in the outer parts of the image. Most areas with larger errors also appear to have high signal posterior standard deviation values (see Figure 9e) and therefore should not be considered as reliable derived estimates. Thus, these reconstruction errors are not only caused by the response approximation error, which happened in the training process discussed earlier, but also due to the fact that the extreme blurring by the synthetic response at the image borders in combination with the Poisson shot noise causes reconstruction ambiguities in the signal space. These ambiguities lead to a larger error and higher standard deviation of the posterior.

In order to see that the algorithm has converged and is working correctly, one can also compare the data (not shown) with the reconstructed signal response (see Figure 9d) by looking at their difference (see Figure 9f). In summary, it can be seen that the efficient response representation can be successfully applied in imaging algorithms.

## 9. Discussion

The need for efficient response representations in imaging led to the development of the models presented in this work, which were inspired by earlier research on butterfly matrices [11]. The efficient structure of butterfly matrices, inherited of fast Fourier transforms (FFT), results in a sub-quadratic algorithm scaling with O(NlogN) that is capable of representing an expensively simulated synthetic response up to 1% error. To this end, ${\mathrm{Net}}_{4}$, a butterfly convolutional network with three butterfly convolution operators (BCOs) in series, non-mirrored architecture, and flat application is used, which is differentiable and thus suitable for the application as a response in generative models for measurement data. Furthermore, we could show that by upscaling the network to a higher resolution the accuracy does not suffer while the representation becomes, as expected, sparser compared to a full matrix representation. This network is also successfully used as a response representation for a spatially variant point spread function (PSF) in an imaging example with synthetic generated photon count data following Poisson statistics. We expect the butterfly network representation to give comparably good results for spatially varying PSFs of real instruments such as Chandra or eROSITA. Although these are more complex, and radially asymmetric, this can be argued because the radial symmetry of the synthetic example is not part of the butterfly transform parameterization. In addition, typical PSFs from real instrument are smaller and therefore sparser than the synthetic PSFs in this work and therefore require fewer degrees of freedom. We also see in our example that most of the approximation error is made in the outer regions of the PSFs, indicating that the error also becomes smaller for smaller PSFs.

To improve the computational performance through GPU support and parallelization, more advanced machine learning platforms such as TensorFlow [25] or PyTorch [26] could be considered. After sufficient training, the corresponding butterfly network can be used to perform high-fidelity imaging using information field theory and NIFTy. Additionally, other fields of application with a connection to slightly inhomogeneous processes are imaginable. These also include time- or energy-dependant processes with variable correlations. All in all, the method to represent instrument response functions introduced in this work is promising to improve imaging with complex photographic instruments and thus should be considered in further research.

## Figures and Tables

**Table 1:** Comparison of the number of parameters needed for a 2D and 1D butterfly transform.

Name	#θ	#ϕ	#γ
2D (m×l grid)	$\frac{m}{2}\log_{2}m+\frac{l}{2}\log_{2}l$	$ml\log_{2}m$	$ml\log_{2}m$
flat (ml)	$\frac{ml}{2}\log_{2}ml$	$ml\log_{2}ml$	$ml\log_{2}ml$

**Table 2:** Parameters and results for all seven network architectures. The density is here defined as the ratio of the number of parameters and the number of entries in a full matrix representation (16${}^{4}$ = 65,536). A lower density indicates a higher efficiency of the representation.

Network Name	${\mathrm{Net}}_{1}$	${\mathrm{Net}}_{2}$	${\mathrm{Net}}_{3}$	${\mathrm{Net}}_{4}$	${\mathrm{Net}}_{5}$	${\mathrm{Net}}_{6}$	${\mathrm{Net}}_{7}$
Number of BCOs	1	2	3	3	3	3	3
Architecture	mr	mr	mr	nmr	mr	nmr	nmr
Design	flat	flat	flat	flat	2D	2D	flat
Likelihood	serial	serial	serial	serial	serial	serial	parallel
$\hat{\zeta }$ in %	7.96	3.14	2.00	1.04	2.45	1.50	6.86
Number of parameters	5632	11,264	16,896	32,256	7872	14,208	32,256
Density in %	8.59	17.19	25.78	49.22	12.01	21.68	49.22

**Table 3:** Comparison of the same network structure applied to different resolutions. The percentage error is on the same scale while the density decreases with higher resolution.

Network Name	Resolution	Number of Parameters	Density in %	$\hat{\zeta }$ in %
${\mathrm{Net}}_{4}\left(16,16\right)$	16×16	32,256	49.22	1.04
${\mathrm{Net}}_{4}\left(32,32\right)$	32×32	159,744	15.23	1.00

**Table 4:** Comparison execution times of one butterfly convolution and a matrix-vector multiplication.

Resolution	Butterfly Convolution		Matrix-Vector Multiplication	
	Time	Memory	Time	Memory
2×2	0.564 ms±4.8 μs	1.84 kB	2.23 μs±1.05 μs	248 B
4×4	1.063 ms±5.7 μs	5.5 kB	1.99 μs±0.66 μs	2.17 kB
8×8	1.59 ms±16 μs	20.3 kB	2.76 μs±0.80 μs	32.9 kB
16×16	2.23 ms±5.58 μs	91.2 kB	12.5 μs±1.6 μs	524 kB
32×32	3.22 ms±142 μs	432.4 kB	298 μs±19 μs	8.4 MB
64×64	5.74 ms±12.27 μs	2.0 MB	3.94 ms±133 μs	134 MB
128×128	16.1 ms±33.0 μs	9.4 MB	0.101s±36.5 μs	2.14 GB
256×256	73.0 ms±92.4 μs	43.0 MB	1.63s±0.90 ms	34.4 GB
512×512	315.6 ms±581 μs	193.0 MB	n.a.	550 GB
1024×1024	1.69 s±2.32 ms	855.6 MB	n.a.	8.8 TB

## Data Availability

Not applicable.

## References

[1] Predehl P., Andritschke R., Arefiev V., Babyshkin V., Batanov O., Becker W., Böhringer H., Bogomolov A., Boller T., Borm K. (2020). The eROSITA X-ray telescope on SRG. arXiv.

[2] Weisskopf M.C., Tananbaum H.D., Van Speybroeck L.P., O’Dell S.L. Chandra X-ray Observatory (CXO): Overview. Proceedings of the X-Ray Optics, Instruments, and Missions III. International Society for Optics and Photonics.

[3] Selig M., Bell M.R., Junklewitz H., Oppermann N., Reinecke M., Greiner M., Pachajoa C., Enßlin T.A. (2013). NIFTY–Numerical Information Field Theory-A versatile PYTHON library for signal inference. Astron. Astrophys..

[4] Steininger T., Dixit J., Frank P., Greiner M., Hutschenreuter S., Knollmüller J., Leike R., Porqueres N., Pumpe D., Reinecke M. (2019). NIFTy 3–Numerical Information Field Theory: A Python Framework for Multicomponent Signal Inference on HPC Clusters. Ann. Phys..

[5] Arras P., Baltac M., Ensslin T.A., Frank P., Hutschenreuter S., Knollmueller J., Leike R., Newrzella M.N., Platz L., Reinecke M. (2019). Nifty5: Numerical Information Field Theory v5.

[6] Enßlin T.A., Frommert M., Kitaura F.S. (2009). Information field theory for cosmological perturbation reconstruction and nonlinear signal analysis. Phys. Rev. D.

[7] Enßlin T. (2014). Astrophysical data analysis with information field theory. Proceedings of the AIP Conference Proceedings.

[8] Enßlin T. (2013). Information field theory. Proceedings of the AIP Conference Proceedings.

[9] Enßlin T.A. (2019). Information theory for fields. Ann. Phys..

[10] Polcari J., Ruffa A., Toni B. (2018). Generalizing the Butterfly Structure of the FFT. Advanced Research in Naval Engineering.

[11] Dao T., Gu A., Eichhorn M., Rudra A., Ré C. Learning fast algorithms for linear transforms using butterfly factorizations. Proceedings of the International Conference on Machine Learning, PMLR.

[12] Alizadeh K., Farhadi A., Rastegari M. (2019). Butterfly Transform: An Efficient FFT Based Neural Architecture Design. arXiv.

[13] Howard A.G., Zhu M., Chen B., Kalenichenko D., Wang W., Weyand T., Andreetto M., Adam H. (2017). Mobilenets: Efficient convolutional neural networks for mobile vision applications. arXiv.

[14] Sandler M., Howard A., Zhu M., Zhmoginov A., Chen L.C. Mobilenetv2: Inverted residuals and linear bottlenecks. Proceedings of the IEEE Conference on Computer Vision and Pattern Recognition.

[15] Howard A., Sandler M., Chu G., Chen L.C., Chen B., Tan M., Wang W., Zhu Y., Pang R., Vasudevan V. Searching for mobilenetv3. Proceedings of the IEEE/CVF International Conference on Computer Vision.

[16] Singhal U., Stella X.Y. Complex-valued Butterfly Transform for Efficient Hyperspectral Image Processing. Proceedings of the 2022 International Joint Conference on Neural Networks (IJCNN).

[17] Lin R., Ran J., Chiu K.H., Chesi G., Wong N. (2021). Deformable butterfly: A highly structured and sparse linear transform. Adv. Neural Inf. Process. Syst..

[18] Song J., Lee Y.H. (2021). Optical image encryption using different twiddle factors in the butterfly algorithm of fast Fourier transform. Opt. Commun..

[19] Eberle V., Frank P., Stadler J., Streit S., Enßlin T. (2022). Efficient Representations of Spatially Variant Point Spread Functions with Butterfly Transforms in Bayesian Imaging Algorithms. Phys. Sci. Forum.

[20] Cooley J.W., Tukey J.W. (1965). An algorithm for the machine calculation of complex Fourier series. Math. Comput..

[21] Wolberg G. (1988). Fast Fourier Transforms: A Review.

[22] Knollmüller J., Enßlin T.A. (2019). Metric Gaussian Variational Inference. arXiv.

[23] Frank P., Leike R., Enßlin T.A. (2021). Geometric variational inference. Entropy.

[24] Nocedal J., Wright S. (2006). Numerical Optimization.

[25] Abadi M., Agarwal A., Barham P., Brevdo E., Chen Z., Citro C., Corrado G.S., Davis A., Dean J., Devin M. (2015). TensorFlow: Large-Scale Machine Learning on Heterogeneous Systems. tensorflow.org.

[26] Paszke A., Gross S., Massa F., Lerer A., Bradbury J., Chanan G., Killeen T., Lin Z., Gimelshein N., Antiga L., Wallach H., Larochelle H., Beygelzimer A., d’Alché-Buc F., Fox E., Garnett R. (2019). PyTorch: An Imperative Style, High-Performance Deep Learning Library. Advances in Neural Information Processing Systems 32.

